# 

**Published:** 1895-03-23

**Authors:** 


### CONTENTS.

**Workshops and Drink** - - - - 433

**Dr. Klein on Antitoxin** - - - - 434

**Treatment and the Pulmonary Circulation** - - - 435

**Modern Sociology.**—Facts about Families - - - 436

**Notes and News** - - - - 438

**Medical Progress and Hospital Clinics:**

The Vaccination Question - - - - 439

Chronic Cerebral Abscess - - - - 441

PROGRESS IN MEDICINE.—Diseases of the Circulatory System - 441

PROGRESS IN SURGERY.—Abdominal Surgery - - - 443

NEW APPLIANCES AND THINGS MEDICAL - - - 444

**Annotations.**—Commercialised Physic: A Protest—Dampness and Rheumatism—Climate and Death-rate - - - 437

**The Institutional Workshop:**

HOSPITAL ADMINISTRATION.—The Planning of Fever Hospitals - 445

THE STORY OF THE INSANE FROM YEAR TO YEAR - - - 445

PRACTICAL DEPARTMENTS.—Heating and Ventilation - - 446

HOSPITAL FESTIVALS AND MEETINGS - - - 447

**Editor's Letter-Box** - - - - 448

**Scraps and Gleanings** - - - - 448

**The Book World of Medicine and Science** - - - 449

**The Student of Medicine** - - - - 449

### EXTRA SUPPLEMENT.—THE NURSING MIRROR.

**News from the Nursing World.**—A Visit to St. Mary's Hospital—Royalty at Aldershot—London Hospital Probationers—Probationers in Fever Hospitals—The Birmingham Needlework Guild—Not "A Washer"—A Little Dispensing—Nurses as Almoners—Hinckley Cottage Hospital—Hindu Bathers—Venezuelan Red Cross Society—East End Mother's Home—Training at Lambeth Infirmary—The North London Nursing Association—Cordial Co-operation - clxxxiii

**Lewisham Infirmary** - - - - clxxxiii

**East London Nursing Society** - - - clxxxiv

**Presentations** - - - - clxxxvi

**The Royal National Pension Fund for Nurses** - clxxxvi

**Appointments** - - - - clxxxvi

**Royal British Nurses' Association** - - - clxxxvii

**Notes and Queries** - - - - clxxxviii

